# Evaluating economic efficiency of the national high-tech industrial development districts in the Yangtze River Delta by stochastic frontier analysis

**DOI:** 10.1016/j.heliyon.2024.e30128

**Published:** 2024-04-20

**Authors:** Chao Yang

**Affiliations:** a School of Public Policy and Management, China University of Mining and Technology, Xuzhou, 221116, China; b School of Safety Engineering, China University of Mining and Technology, Xuzhou, 221116, China

**Keywords:** Economic efficiency, Regional innovation capability, Innovation district, Sustainable development

## Abstract

The High-tech Industrial Development Districts (HIDDs) are technological engines for the regional economy in China. The Chinese government implemented the Torch Initiative to accelerate industrial agglomeration and innovation development by administratively upgrading the local HIDDs into a national list since 1989. The policy intervention emphasizes the labor and capital inputs on science and technology. The study adopts the Stochastic Frontier Analysis (SFA) to understand the economic efficiency of the enlisted national HIDDs in the Yangtze River Delta. The results suggest that the average efficiency trends of the all-in-total sales, product sales and commodity sales are decreasing while that of technology sales is increasing from 2007 to 2019. In the total sales efficiency evaluation, most early-enrolled HIDDs are in the high-efficiency group whereas the newcomers are in the low-efficiency group. The Nantong HIDD has the highest efficiency, followed by Wuxi, Taizhou, Suzhou, Nanjing, Shanghai ZJ, Xiaoshan, Ningbo, Suzhou IP and Jiangyin HIDDs. Huainan, Huaian, Tongling, Shanghai ZZ, Lianyungang and Suqian HIDDs rank very low in the total sales evaluation. Besides, Hangzhou, Nantong and Ningbo HIDDs rank first in the technology, product and commodity sales efficiency evaluation correspondingly. In addition, the increase of S&T labor inputs would decrease the efficiency of the product, commodity and total sales while the increase of the S&T capital inputs is as expected a facilitator to the efficiency of product, commodity and total sales. This study contributes to the discussion on the role of political intervention in technological innovation by breaking down the economic efficiency into three major parts, the efficiency of technology, product and commodity sales. The findings could help policymakers strike a balance between the human and capital inputs in regional innovation capability.

## 1 Introduction

High-technology Industrial Development Districts (HIDDs) serve as an economic engine for China. In 2020, the total business revenue of these HIDDs in China is 6.72 trillion USD, accounting for 45.65 % of the country's Gross Domestic Product. This is mainly attributed to the Torch Initiative by the Ministry of Science and Technology of China (MOST). Like many countries to copy the success of the Silicon Valley model in the United States, this program aims to realise industrial agglomeration through government policy [[1], [2], [3]]. The Torch Initiative was established in 1989 to propel the high-tech sectors of the whole country. It is under the arrangements of this initiative that the regional high-tech industry district could be upgraded to the national list if it fulfils the requirements. There were 190 provincial HIDDs upgraded to national HIDDs across the country by 2022. This “upgrading” policy aims to boost the scientific and technological base while also speeding up technology transfer and entrepreneurship [4]. Although it seems only an administrative hierarchy upward for a HIDD, the enlisted HIDD would enjoy exclusive innovation policies, such as tax incentives, rent concessions, innovation vouchers, etc. It is the most important science and technology policy and has been implemented for more than 35 years. It is to dissolve the dilemma of ineffective transferring from scientific research to economic development. As a result, the enlisted competition among the local and regional HIDDs is very fierce.

Previous research on the HIDDs in China has explained the interaction between high-tech industries and the mobilization of urban resources. First, the spatial distributions of HIDDs have great impacts on the urban economy and innovation system. The upgraded HIDDs are pilot regions in applying new policy tests and pushing forward the innovation capability of the cities and their neighboring regions [5]. The HIDD upgrading policy enhances innovation cooperation, innovation spillover, and local economic performance [5,6]. The high-tech industries have long been concentrated geographically but low intraregional specifications [7]. The spatial network of the innovation correlation is not connected very closely but eastern China is better than western China in terms of innovation connectedness [8]. It also suggests that the eastern coastal HIDDs become one of the innovation cohesion subgroups [7,8]. The comprehensive efficiency of national HIDDs is better than that of provincial HIDDs and economic-technological development zones (EDZs) in an underdeveloped province of China [9].

Second, the development of national HIDDs affects land use efficiency. The nonlinear grey Bernoulli model was applied to predict the employment demand of an HIDD [10]. An interesting finding is that the university science park could support high employment of a national HIDD but this phenomenon disappeared after 2000 [11]. A case study of Wuhan Donghu HIDD reveals systematic interaction among the governments, enterprises, universities and intermediaries, especially the close relationship between innovation and entrepreneurship [12]. HIDDs speed up urbanization through spatial production under the arrangement of government policy [13]. For example, firms in a Shanghai HIDD were found to be more efficient in land use than those located outside the district [14]. The ecological impact of the land use change is also measured by a case study on the Nanchang High-tech Development Zone [15]. Overall, the newly enlisted national HIDDs have positive impacts on land use efficiency and urban development [16].

Third, HIDDs are government-led industrial parks where the science and technology policies under the Torch initiative create a good business environment. For example, Shenyang Tiexi Industrial Zone, located in Northeastern China, cancelled the institutional barrier and enhanced the private economy under the guidance of the governments [17]. The national HIDDs attracted a great number of transnational human capital, including foreign PhDs and domestic returnee scholars [18]. The case of Guangzhou HIDD reveals that the administrative upgrading would improve the industrial structures and urban economic competitiveness [19]. The case of Hefei HIDD shows that industrial policies are the most important external sources affecting the acquisition strategies of enterprises [20]. But some attribute the lack of innovation in China to political interventions although they agree that the HIDD policy aims to make China become the innovation center of the world [21]. It was found that foreign direct investment promotes the growth of HIDDs but has no significant impact on innovation efficiency [22].

The input-output efficiency evaluation could provide a new perspective on this question. We explain the economic performance of national HIDDs from three sales outputs, technological, product and commodity sales rather than the comprehensive efficiency evaluation. A national HIDD with better comprehensive efficiency does not necessarily prove its advantage in innovation in the high-tech industry. The main contribution of this study is to understand the economic efficiency of the Chinese national HIDDs from the input-output analysis. The evaluation deepens the understanding of the impacts of the national HIDD upgrading policy by evaluating the technology efficiency, production efficiency and commodity efficiency. This research extends the previous literature on the innovation development of the high-tech industry in China [9,[21], [22], [23], [24], [25]].

We chose Yangtze River Delta (YRD) as the study area for the following reasons. First, the Yangtze River Delta region, which includes megacities like Shanghai, Nanjing, and Hangzhou, is one of China's most technologically advanced manufacturing regions, with 34 national HIDDs enlisted in the Torch initiative. Second, the development of the Yangtze River Delta is unbalanced, including developed and underdeveloped provinces. Third, the Yangtze River Delta Integration Plan was first implemented in 2010 and updated in 2019. The first phase of integration was from 2010 to 2015. This plan adopts a more holistic approach to science and industrial innovation, public infrastructure, urban cooperation and public services. This research also corresponds to this policy plan to reveal the high-tech industry efficiency differences inside the YRD in the period. Hence, it also contributes to the literature on the evaluation of the high-tech industry development in the Yangtze River Delta [[26], [27], [28], [29]].

Efficiency evaluation on the HIDDs arranges how well the production is. The optimal inputs and outputs are the objectives for the management teams and many other stakeholders, including local and central government officials, investors, enterprises and so on. That is, how to make good use of inputs and attain the largest outputs could be relatively fair in considering the question. Many previous researches use Data Envelopment Analysis (DEA) as the evaluation method to find the efficient and inefficient decision-making units [24,30], but this method is deterministic and the deviation from the best practice would be inefficiency. Stochastic Frontier Analysis treats the deviation as both random error and inefficiency [31]. We take this approach to evaluate the economic efficiency of 34 national HIDDs in the Yangtze River Delta to rank the efficiency of technology sales, product sales, commodity sales and total sales from 2007 to 2019 to help understand the regional innovation system.

The remainder of the article is organized as follows. Section 2 describes the materials and methods in the study, including the methods, data sources, and variable description. Section 3 reports the results of the empirical analysis before a discussion is provided. Section 4 is the conclusion.

## 2 Materials and methods

### 2.1 Stochastic frontier analysis

The seminal work by Farrell proposes a satisfactory method to evaluate productive efficiency based on the input and output [32], which introduces data envelopment analysis (DEA). This work ignites the interest of the academy to the production frontiers and technical efficiency. It was suggested that the estimation of frontier production could be better when using stochastic frontier analysis [33]. Meeusen and Broeck [34] provide an efficiency model with a statistical disturbance due to randomness. The advantage of the method is to identify the specification of the error term in two separate parts, that is, normal and one-sided distributions. These literature lays a foundation for the SFA for cross-sectional data. Then, Schmidt and Sickles provide various estimators and develop an SFA extension for panel data [35]. But this extension is assumed on a time-invariant basis. According to Ref. [35], the stochastic frontier model is expressed as equation (1).

(1) $$y_{it}=\alpha +x_{it}\beta +v_{it}‐u_{i}\;\;(i=1,2,\ldots ,n;\;t=1,2,\ldots ,T)$$

Where y_it_ is the logarithm of the output,

x_it_ is the vector of inputs in the ith individual in t time,

v_it_ is the regular disturbance

u_i_ is the nonnegative random variables and represents technical efficiency.

And the technical inefficiency estimation formula is equation (2).

(2) $${\hat{u}}_{i}=\max\left({\hat{\alpha }}_{i}\right)-{\hat{\alpha }}_{i}\geq 0\;(i=1,2,\ldots ,n)$$

Battese and Coelli provide a model for the time-varying effects for unbalanced panel data [36]. Hence, we have equations (3), (4).

(3) $$Y_{it}=f\left(x_{it},\beta \right)\exp\left(V_{it}-U_{it}\right)$$

(4) $$U_{it}=\eta_{it}U_{i}=\left\{\exp\left[-\eta \left(t-T\right)\right]\right\}U_{i},\;t\in \tau \left(i\right),\;(i=1,2,\ldots ,N)$$

Where Y_it_ is the observations of output of the firm,

f(x_it_, β) is the function of inputs for the ith firm in the tth time,

V_it_ and U_it_ are the random errors and truncations respectively,

η represents an unknown parameter,

τ(i) is the time set of the ith firm. And the model is half-normal.

Note that $U∼N\left[0,\sigma_{u}^{2}\right]$ and $V∼N\left[0,\sigma_{v}^{2}\right]$ and σ is the standard deviation parameter.

And following the translog SFA model [37,38], the model could be written as equation (5).

$$\ln\;Y_{it}=\beta_{0}+\beta_{1}\;\ln\;Lab+\beta_{2}\;\ln\;Cap+\beta_{3}\;\ln\;t+\beta_{4}\;\ln\;Cap\;\ln\;Cap+\beta_{5}\;\ln\;Lab\;\ln\;Lab+\beta_{6}\;\ln\;t\;\ln\;t+\beta_{7}\;\ln\;Cap\;\ln\;Lab+\beta_{8}\;\ln\;Cap\;\ln\;t+\beta_{9}\;\ln\;t\;\ln\;Lab+v_{it}-u_{it}$$

(5) updatedintotheaboveformula

Where Cap stands for capital input, Lab for labor, and t for time.

In the current study, the Cobb-Douglas and translog SFA models are applied since they can help better understand efficiency. The advantage is that the cross-relationships and non-linearities in this model might be recognized by the second-order components [39]. The hicks-neutral model and time-variant or invariant model are also applied with reference to Mizobuchi [40] and Cowie [41]. We employ the Frontier 4.1 application, the most frequently used application invented by Tim Coelli, to calculate the efficiency in our study [36,42]. The application can be found at the page of the Center for Efficiency and Productivity Analysis, The University of Queensland, Austrilia (https://economics.uq.edu.au/cepa/software).

### 2.2 Data and variables

YRD is the most technologically advanced urban agglomeration area, encompassing Shanghai, Jiangsu, Zhejiang and Anhui provinces. There are 34 national HIDDs in the YRD area by 2022, when there were only 9 in 2007. This leads to an unbalanced panel dataset from 2007 to 2020. The observations are 280. We chose 2007 as the starting year of the data since this is the first year the Torch program released the statistical yearbook in 2008. SFA is applicable of dealing with the unbalanced panel data. The 34 national HIDDs are Shanghai Zhangjiang (ZJ), Shanghai Zizhu (ZZ), Nanjing, Wuxi, Jiangyin, Xuzhou, Changzhou, Wujin, Suzhou HIDD, Suzhou Industrial Park (Suzhou IP), Kunshan, Changshu, Nantong, Lianyungang, Huaian, Yancheng, Yangzhou, Zhenjiang, Taizhou, Suqian, Hangzhou, Xiaoshan, Ningbo, Wenzhou, Jiaxing, Moganshan, Shaoxing, Quzhou, Hefei, Wuhu, Bengbu, Huainan, Maanshan and Tongling. They are distributed across Shanghai, Jiangsu, Zhejiang, and Anhui Provinces, accounting for 20.1 percent of the total number of HIDDs in China and 26.5 percent of the total industrial output value. All of these figures come from the China Torch Statistical Yearbooks.

Table 1 is the variable definitions and selection criteria. The input variables include Science and Technology (S&T) Spending, the quantity of S&T employees and time (t). Science and technology spending refers to the money spent on S&T activities and the managerial inputs that go along with them. This expenditure only covers the expenses for research and development instead of actual production. All labor who follows a scientific and technology profession in a national HIDD is counted as S&T personnel. Another input is time cost, which is the time length from when a HIDD was enlisted in the program. The output variables are the annual sales turnover of technology, product and commodity, and all-in-total in a HIDD respectively. The annual sales turnover of technology consists of the turnover from technology consultation and service, technology transfer and contracting, technology as capital investment, pilot technology and technology-related business in one year. The annual sales turnover of products covers sales revenue from the manufactured and semi-made products, and labor services in one year. The annual sales turnover of commodities are mainly from selling products manufactured by other enterprises in one year. The annual sales turnover of all-in-total is comprised of the above three sales turnovers in a year.

**Table 1:** Variables definitions and selection criteria.

Variables (Acronym)	Variable Definition	Unit	Selection Criteria
S&T personnel (lnLab)	The logarithm value of labor employed in the science and technology vocations in a HIDD in a year	Person	On the basis of [9,43,44]
S&T spending (lnCap)	The logarithm value of capital investment in the science, technology and innovation in a HIDD in a year	1,000 Yuan	On the basis of [45]
Time (lnt)	The logarithm value of the time length of upgrading to a national HIDD	Year	Time cost
Technology Sales (OutputT)	The logarithm value of sales turnover of technology transfer, licensing, counselling and services in a national HIDD	1,000 Yuan	On the basis of [6,9,30]
Product Sales (OutputP)	The logarithm value of sales turnover of finished and semi-finished products and services in a national HIDD	1,000 Yuan	
Commodity Sales (OutputC)	The logarithm value of sales turnover of selling manufactured products which are bought from other enterprises in a national HIDD	1,000 Yuan	On the basis of [6,9,30]
All-in-total Sales (OutputA)	The logarithm value of the total sales turnover of the technology, product and commodity sales	1,000 Yuan	On the basis of [6,9,30]

All the data are on the HIDD level. We choose S&T-related variables based on that the policy aim of the Torch initiative is to improve the science, technology, and innovation capability of the industry. Therefore, all the efficiency evaluations are based on S&T inputs rather than on all the resource inputs. The descriptive statistics of the variables is in the supplementary material.

## 3 Results and discussion

This paper used stochastic frontier analysis models to investigate the efficiency of HIDDs in the YRD area from 2007 to 2019. We build models for each output variable to see if a Cobb-Douglas or translog frontier production function is appropriate (models 1 and 2), if there is technical change (model 3), if the technical change is hicks-neutral (model 4), and if technical changes over time (model 5). The output variables of technology, product and commodity sales turnover are tested. The final maximum-likelihood estimates are included in the tables.

### 3.1 The technology sales efficiency evaluation and rankings of HIDDs

Table 2 presents the SFA result on the efficiency of technology sales motivated by S&T inputs. The log-likelihood ratio (LR) test was used for the model comparison test. It is calculated by −2*[*L(H*_*0*_*)-L(H*_*A*_)], where *L(H*_*0*_*)* and *L(H*_*A*_*)* are the log-likelihood function (LLF) values in the results of the models [46]. In this study, *L(H*_*A*_*)* is the log-likelihood function values of the model 2. For example, when calculating the LR of model 1, *L(H*_*0*_*)* is the LLF of model 1. According to the above formula, the LR of the model 1 is 3.832 (<7.05). The LR test results here reveal that the Cobb-Douglas form is better than the translog form, though the gamma in both models is significant at 1 % level. The gamma in model 1 is 0.528, indicating that there exists an inefficiency effect in technical efficiency evaluation. The variables of S&T employees and S&T spending are significant with coefficients of 0.651 and 0.562, respectively. This reflects the positive impacts on the technology sales in the national HIDDs. This is consistent with the findings that labor input is a determinant for HIDD development [5]. In addition, the σ^2^ of model 1 is significant at 1 % level. This reveals the S&T inputs have positive and significant impacts on the technology sales turnover of a national HIDD. Since the LR of model 3 is −2.734 (<7.05), it reflects there is no technical change. The LR of model 4 is −3.047, indicating that the technical efficiency is hicks-neutral. That is, the labor-to-capital ratio remains constant. The technical efficiency varies over time based on that the LR of model 5 is 13.3 (>5.14).

**Table 2:** The results of SFA models on the technology sales turnover.

Variables	Model 1	Model 2	Model 3	Model 4	Model 5
β_0:_constant	1.823 (0.843)	−4.176 (−0.253)	−9.104 (−0.596)	−7.641 (−0.488)	−16.951 (−1.017)
β_1:_lnLab	0.651** (2.371)	−3.301 (−0.629)	−3.869 (−0.826)	−3.736 (−0.799)	−5.631 (−1.030)
β_2:_lnCap	0.562** (1.973)	3.512 (0.647)	4.866 (1.023)	4.557 (0.950)	6.168 (1.122)
β_3:_lnt	−0.016 (−0.404)	0.360 (0.581)		−0.004 (−0.039)	1.145*** (2.136)
β_4:_ (lnCap)^2^		−0.534 (−1.137)	−0.528 (−1.444)	−0.529 (−1.426)	−0.544 (−1.101)
β_5:_ (lnLab)^2^		−0.909 (−1.579)	−0.725* (−1.680)	−0.771* (−1.711)	−0.675 (−1.120)
β_6:_ (lnt)^2^		−0.001 (−0.228)		−0.001 (−0.205)	0.002 (0.398)
β_7:_lnCap*lnLab		1.414 (1.423)	1.218 (1.622)	1.262* (1.640)	1.241 (1.179)
β_8:_lnt*lnCap		−0.014 (−0.166)			−0.100 (−1.185)
β_9:_lnt*lnLab		−0.015 (−0.179)			0.048 (0.558)
σ^2^	1.515*** (4.028)	1.473*** (3.936)	1.579*** (4.325)	1.502*** (4.067)	2.005*** (4.132)
γ	0.528*** (4.418)	0.518*** (4.087)	0.553*** (5.127)	0.529*** (4.439)	0.615*** (6.181)
LLF	−395.663	−393.747	−394.296	−394.140	−402.310

Note: the value in brackets is the t-ratio, which is the ratio between coefficient and standard error. According to the t-ratio, we can apply * to show the level of significance. Single, double and triple stars (*, **, ***) show statistical significance at the level of 10 %, 5 % and 1 %, respectively.

**Fig. 1:**
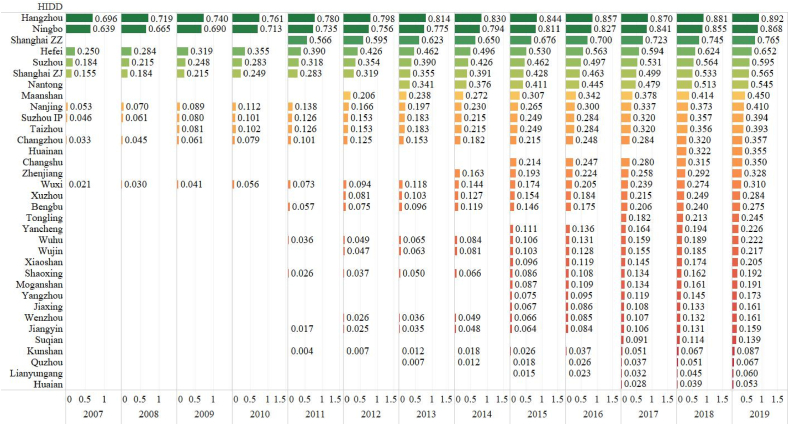
The HIDD Efficiency Ranking of Technology Sales (2007–2019) Source: Own Note: The numbers in the figure are efficiency values of the Cobb-Douglas Function.

Fig. 1 illustrates the technology sales efficiency based on the Cobb-Douglas function. The rankings show the huge difference across the regions. The top 10 high-efficiency HIDDs are Hangzhou, Ningbo, Shanghai ZZ, Hefei, Suzhou, Shanghai ZJ, Nantong, Maanshan, Nanjing, Suzhou IP and Taizhou HIDDs while the lowest-efficiency HIDDs are Huaian, Lianyungang, Quzhou, Kunshan and Suqian HIDDs. Shanghai and its surrounding HIDDs are more advantageous than the others in the YRD. An exception in the top 5 is Hefei HIDD, which is not bordering Shanghai. Hefei HIDD ranks third or fourth through the whole period. This supports the previous findings that Suzhou and Hefei have more migrant flows of high-skilled workers in the YRD [8, 28, 47]. The poorest performed HIDDs are located mainly in North Jiangsu. One noticeable finding is that newly enlisted HIDDs are relatively inferior and rank low in these rankings.

### 3.2 The product sales efficiency evaluation and rankings of HIDDs

Table 3 presents the SFA result on the efficiency of product sales. The translog production function (model 2) is better at explaining the efficiency of product sales turnover than the Cobb-Douglas form (model 1) based on the LR value 23.991 (>7.05). In the model 2 and 5, all the variables except the time length have significant explanatory effects on the efficiency. The LR test of model 5 is 2.8 (<7.05), which also indicates the time length does not have a significant impact on the efficiency. In the model 2 and 5, the coefficients of S&T labor have negative signs, meaning the increase of S&T labor input can reduce the technical efficiency of the product sales. On the other hand, the positive sign of the S&T capital in the models suggests the increase of S&T capital could increase the technical efficiency. The results of models 3 and 4 affirm that there exists technical change but not hicks neutral based on the LR test with 26.959 (>7.05) and 20.572 (>7.05), respectively. The LR test of model 5 is 2.8 (<5.14), indicating that the technical efficiency does not change with time. The gamma of all five models is around 0.84 to 0.91 at 1 % significance level. This reflects that random noises play a significant role in technical inefficiency.

**Table 3:** The results of SFA models on the product sales turnover.

Variables	Model 1	Model 2	Model 3	Model 4	Model 5
β_0:_constant	12.020*** (18.758)	7.097* (1.734)	9.366*** (2.955)	9.623*** (2.938)	8.069** (2.052)
β_1:_lnLab	0.460*** (7.033)	−2.796** (−2.109)	−0.753 (−0.672)	−0.099 (−0.093)	−2.472** (−2.016)
β_2:_lnCap	0.164** (2.374)	2.945** (2.176)	1.332 (1.257)	0.804 (0.769)	2.539** (2.057)
β_3:_lnt	0.023*** (2.992)	−0.190 (−1.268)		0.048** (2.535)	−0.131 (−0.954)
β_4:_ (lnCap)^2^		−0.406*** (−3.174)	−0.140 (−1.626)	−0.084 (−1.030)	−0.377*** (−3.308)
β_5:_ (lnLab)^2^		−0.542*** (−3.480)	−0.187* (−1.728)	−0.135 (−1.317)	−0.526*** (−3.728)
β_6:_ (lnt)^2^		−0.004*** (−2.761)		−0.002 (−1.458)	−0.004*** (−2.913)
β_7:_lnCap*lnLab		0.945*** (3.445)	0.319* (1.742)	0.205 (1.193)	0.901*** (3.667)
β_8:_lnt*lnCap		0.072*** (3.567)			0.067*** (3.462)
β_9:_lnt*lnLab		−0.086*** (−4.402)			−0.083*** (−4.396)
σ^2^	0.399* (1.863)	0.227** (2.444)	0.222*** (4.293)	0.348* (1.944)	0.237*** (2.721)
γ	0.910*** (18.417)	0.854*** (14.346)	0.838*** (26.756)	0.897*** (16.462)	0.857*** (16.228)
LLF	5.0349	17.030	3.551	6.745	15.613

Note: the value in brackets is the t-ratio, which is the ratio between coefficient and standard error. According to the t-ratio, we can apply * to show the level of significance. Single, double and triple stars (*, **, ***) show statistical significance at the level of 10 %, 5 % and 1 %, respectively.

**Fig. 2:**
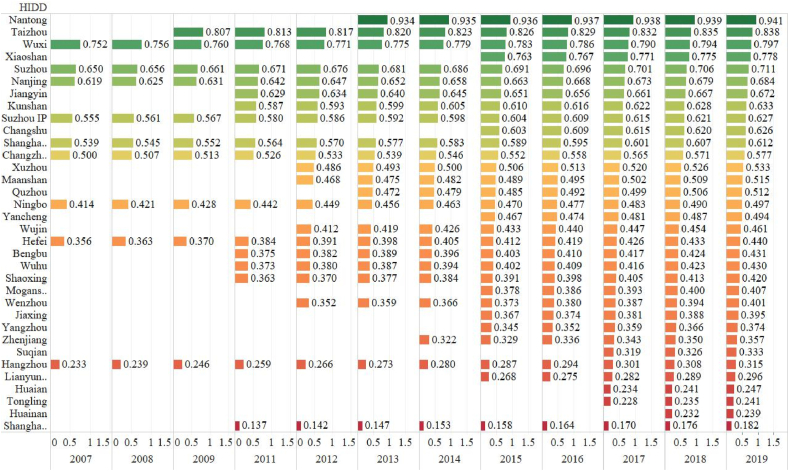
The HIDD Efficiency Ranking of Product Sales (2007–2019) Source: Own Note: The numbers in the figure are efficiency values of translog SFA.

Fig. 2 presents the product sales efficiency ranking of the national HIDDs. This type of efficiency is higher than the technology and commodity sales efficiency in each HIDD on the whole. The landscape of rankings changes dramatically. Surprisingly, Nantong, Taizhou, Xiaoshan, Jiangyin, Kunshan and Changshu HIDDs are among the top 10 high-efficiency districts since these national HIDDs are located in medium-scale cities while Shanghai ZZ, Hangzhou, and Hefei are lower than the average. Shanghai ZZ has the lowest efficiency of all the HIDDs in this figure. The newly enlisted HIDDs such as Nantong and Xiaoshan can also have good performance in this evaluation while some early-enlisted HIDDs are poorly rated, including Shanghai ZZ and Hangzhou HIDDs. This could provide evidence of the view that local governments' competition to upgrade their local HIDDs into the national HIDD list is beneficial to product sales. This extends the previous research [6,43,48] by comparing the efficiency differences between the early-enlisted and the newly-enlisted HIDDs. Enlisting and upgrading into the national list is an important way to show the officials’ political and governance performance.

### 3.3 The commodity sales efficiency evaluation and rankings of HIDDs

Table 4 suggests the results of SFA models on the efficiency of commodity sales. Based on the LR of model 1 is 20.627 (>7.05), we can find that the translog form (model 2) outperforms the Cobb-Douglas form (model 1). In the model 2, the gamma is 0.567, with a significance level of 1 %, indicating that there is technological inefficiency. The gamma value here is much lower than that in the product sales efficiency evaluation, which reveals the role of random noise in the technical inefficiency is also lower. Model 3 omits the time-related variables to test whether the technical change exists. The LR of model 3 is 3.343 (<7.05), demonstrating there is no technical change. Model 4 omits the interaction items such as t*LnCap and t*LnLab to test whether the technical change is hicks-neutral. The LR value of model 4 is 3.028 (<7.05), so it is hicks-neutral. That is, the technical change is not relevant to the inputs. The LR test of model 5 is −0.6 (<5.14), revealing that the technical efficiency is fixed and does not change with time.

**Table 4:** The results of SFA models on the commodity sales turnover.

Variables	Model 1	Model 2	Model 3	Model 4	Model 5
β_0:_constant	2.040 (1.377)	−57.142*** (−57.419)	−59.646*** (−58.816)	−61.187*** (−60.429)	−57.209*** (−57.722)
β_1:_lnLab	0.212 (0.596)	−18.845*** (−21.608)	−23.417*** (−20.137)	−23.820*** (−21.770)	−19.015*** (−21.349)
β_2:_lnCap	0.846*** (2.951)	20.881*** (30.723)	24.306*** (34.426)	24.662*** (37.530)	20.630*** (31.198)
β_3:_lnt	0.034 (0.639)	0.212 (0.3033)		0.137 (1.069)	0.838 (1.124)
β_4:_ (lnCap)^2^		−2.224*** (−10.92)	−2.288*** (−9.1261)	−2.600*** (−13.026)	−2.093*** (−7.885)
β_5:_ (lnLab)^2^		−2.838*** (−5.818)	−2.485*** (−4.3014)	−3.220*** (−6.534)	−2.618*** (−4.351)
β_6:_ (lnt)^2^		−0.005 (−0.685)		−0.004 (−0.514)	−0.004 (−0.566)
β_7:_lnCap*lnLab		4.885*** (7.7346)	4.729*** (6.187)	5.682*** (8.989)	4.575*** (5.843)
β_8:_lnt*lnCap		0.014 (0.1455)			−0.073 (−0.684)
β_9:_lnt*lnLab		−0.028 (−0.304)			0.046 (0.463)
σ^2^	2.644** (2.211)	2.405*** (4.8189)	2.170*** (4.170)	2.392*** (3.2949)	2.873*** (4.311)
γ	0.567*** (5.405)	0.567*** (9.0171)	0.511*** (22.183)	0.547*** (6.843)	0.621*** (10.987)
LLF	−463.953	−453.640	−455.311	−455.154	−453.349

Note: the value in brackets is the t-ratio, which is the ratio between coefficient and standard error. According to the t-ratio, we can apply * to show the level of significance. Single, double and triple stars (*, **, ***) show statistical significance at the level of 10 %, 5 % and 1 %, respectively.

**Fig. 3:**
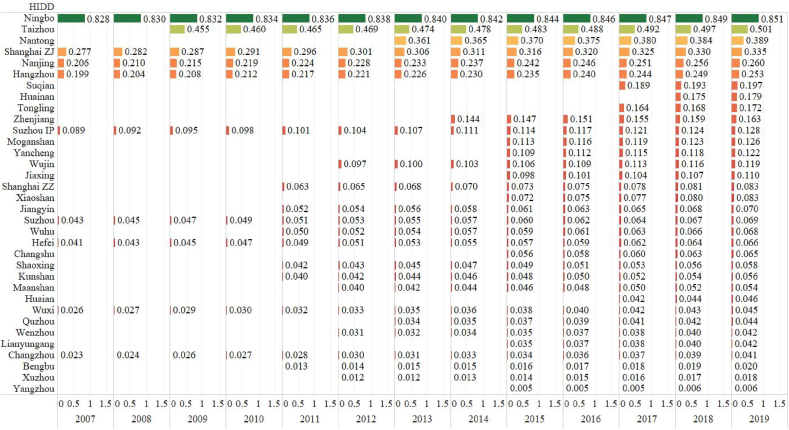
The HIDD Efficiency Ranking of Commodity Sales (2007–2019) Source: Own Note: The numbers in the figure are efficiency values of translog SFA.

Fig. 3 shows the efficiency ranking of commodity sales. It is worth noticing that so many HIDDs are lower than 0.1 in this figure, including Shanghai ZZ, Hefei, Suzhou and Changzhou HIDDs. Only Ningbo HIDD performs very good at more than 0.8 all the years. The impact of the S&T labor and capital on the technical efficiency shows similar impacts with those on the technical efficiency of the product sales based on the signs of β_1_ and β_2_ coefficients in Table 4. The S&T labor and capital investments have the biggest impact on the commodity sales than the technology and product sales. This could explain why commodity efficiency is the least efficient of the three efficiency types. After all, commodity businesses does not play a major role in the outputs of HIDD, which are established for the science, technology and innovation under the Torch initiative. The findings supplement the previous literature on the efficiency evaluation of the high-tech industry by considering the commodity businesses in the S&T-oriented industrial districts [24,49,50].

### 3.4 The all-in-total sales Sales efficiency evaluation and rankings of HIDDs

Table 5 shows the SFA results of the all-in-total sales efficiency evaluation. The gammas in the model 1 and 2 are 0.921 and 0.908, corresponding, which are at 1 % significant level. This reflects the random shocks dominate the technical inefficiency in this situation. Then, the LR test of model 1 is 31.574 (>7.05), indicating the translog form (model 2) is more appropriate than the Cobb-Douglas form in explaining the efficiency. The coefficients of the S&T labor and capital have the same signs as in the models of product and commodity sales efficiency. The negative sign of S&T labor means that the increase in S&T employment would reduce the technical efficiency of the total sales. The positive signs of S&T capital mean that the increase of the S&T investment would improve the technical efficiency of the overall sales. This provides new evidence of the role of S&T employment and investment in the innovation process of the high-tech industry [11,18]. The LR of model 3 is 37.290 (>7.05), which suggests the existence of technical change. Then, the interaction items of lnt*lncap and lnt*lnlab are omitted to test whether it is hicks-neutral. The LR of model 4 is 23.173 (>7.05), indicating the technical change is not hicks-neutral. This finding reflects that S&T labor and capital inputs are relevant to the total sales. The results of model 5 suggest the technical efficiency does not vary over time based on that the LR of model 5 is only 4.8 (<5.14). In both model 2 and 5, the coefficients of lnt*lnt are negative and significant at 1 % level, which reflects the technical change increased at a decreasing rate. The coefficient on the interaction item between time and capital is around 0.07 at 1 % significant level. This reveals that one unit of capital investment increase would bring a 7 % increase rate of the technical change per year on average in the national HIDDs of the YRD region. In contrast, the one-unit increase in S&T labor could decrease by about 8.6 % per year. This finding clarifies the previous research by quantifying the impact of the S&T labor on the total sales efficiency of HIDDs [11].

**Table 5:** The results of SFA models on the all-in-total sales turnover.

Variables	Model 1	Model 2	Model 3	Model 4	Model 5
β_0:_constant	11.846*** (21.55)	9.294** (2.3064)	8.643*** (2.9981)	11.834*** (3.4506)	8.866** (2.262)
β_1:_lnLab	0.488*** (7.819)	−2.498** (−2.199)	−2.092*** (−2.854)	−0.153 (−0.147)	−2.535** (−2.175)
β_2:_lnCap	0.155** (2.319)	2.443** (2.0122)	2.327*** (3.1124)	0.525 (0.5147)	2.431** (2.035)
β_3:_lnt	0.034*** (4.677)	−0.171 (−1.106)		0.074*** (4.0692)	−0.096 (−0.660)
β_4:_ (lnCap)^2^		−0.386*** (−3.593)	−0.247*** (−3.833)	−0.098 (−1.234)	−0.401*** (−3.564)
β_5:_ (lnLab)^2^		−0.554*** (−4.276)	−0.297*** (−2.981)	−0.194* (−1.916)	−0.594*** (−4.111)
β_6:_ (lnt)^2^		−0.006*** (−4.232)		−0.003*** (−2.543)	−0.005*** (−3.776)
β_7:_lnCap*lnLab		0.935*** (4.1492)	0.549*** (3.5902)	0.282* (1.6657)	0.993*** (4.027)
β_8:_lnt*lnCap		0.073*** (3.6707)			0.068*** (3.351)
β_9:_lnt*lnLab		−0.086*** (−4.682)			−0.087*** (−4.450)
σ^2^	0.455* (1.787)	0.344 (1.607)	0.217*** (5.0584)	0.436 (1.5438)	0.216** (2.388)
γ	0.921*** (20.288)	0.908*** (15.275)	0.837*** (30.043)	0.921*** (17.472)	0.848*** (12.824)
LLF	7.301	23.090	4.445	11.504	20.676

Note: the value in brackets is the t-ratio, which is the ratio between coefficient and standard error. According to the t-ratio, we can apply * to show the level of significance. Single, double and triple stars (*, **, ***) show statistical significance at the level of 10 %, 5 % and 1 %, respectively.

**Fig. 4:**
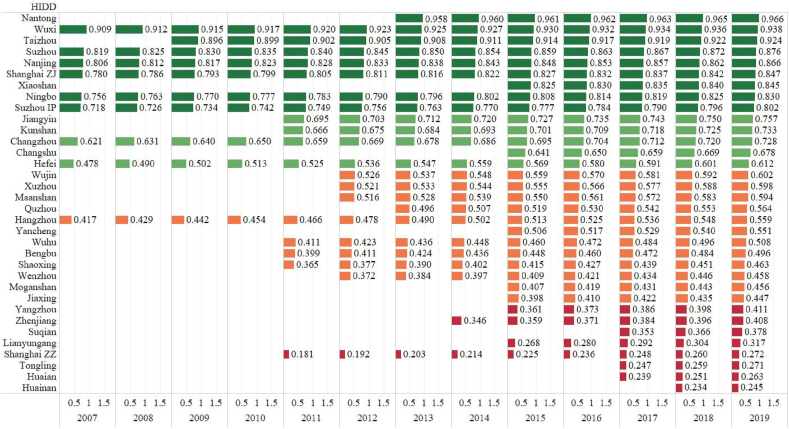
The HIDD Total Sales Efficiency Ranking of All-in-total Sales (2007–2019) Source: Own Note: The numbers in the figure are efficiency values of translog SFA.

Fig. 4 illustrates the all-in-total sales efficiency ranking in the YRD. These national HIDDs are distributed evenly at every 0.1 range. It suggests that there is a lot of room for growth in the future. Most early-enrolled HIDDs are in the high-efficiency group (Efficiency value > 0.6) whereas those in the low-efficiency group (<0.4) are newcomers to the national HIDD list. This could be linked to the maturity of an HIDD's innovation system. The Nantong HIDD has the highest efficiency, followed by Wuxi, Taizhou, Suzhou, Nanjing, Shanghai ZJ, Xiaoshan, Ningbo, Suzhou IP and Jiangyin HIDDs. Huainan, the latest upgraded national HIDD in our research, has the lowest efficiency. Another intriguing result is that the efficiency rankings of the two HIDDs in Shanghai are different, with the Shanghai ZJ rating sixth from the top and the Shanghai ZZ ranking third from the bottom. This is most likely due to the fact that the Shanghai ZZ HIDD excels at technology sales efficiency but lags in product and commodities sales efficiency. This new finding complements the earlier studies by recognizing the efficiency differences of the two HIDDs in Shanghai [26,27,51].

### 3.5 The overall trends of average efficiency change of the HIDDs

**Fig. 5:**
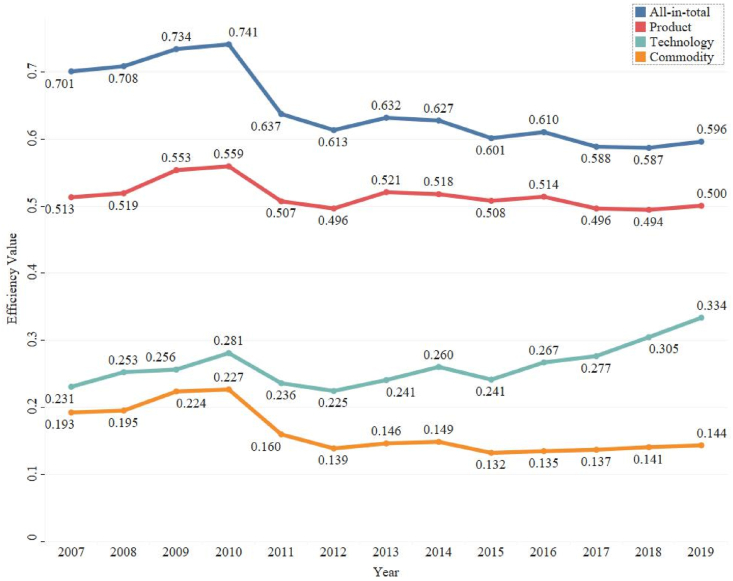
The average S&T-motivated economic efficiency of 34 national HIDDs in the YRD Source: Own Note: The numbers in the figure are average efficiency values.

Fig. 5 presents the average economic efficiency of 34 national HIDDs in the YRD, including the efficiency of technology, product, commodity and all-in-total sales. Firstly, the average efficiency of all-in-total sales, product sales and commodity sales is going downward while the average efficiency of technology sales is increasing from 2007 to 2019. Secondly, The all-in-total sales average efficiency has been steadily declining for 13 years. It climbs to 0.741 in 2010, then drops sharply to 0.6 in 2012 and keeps this number around the year after. The efficiency trend of all-in-total sales is very similar to that of product sales, but the difference is that the average efficiency is much higher. Thirdly, among the breakdown efficiency evaluation, the average efficiency of product sales is the highest. It is almost stable with a gentle drop at the end, from 0.513 in 2007 to 0.500 in 2019. Fourthly, the average efficiency of technology sales is in the middle between that of product and commodity. The average efficiency of technology sales turnover did not increase very fast from 0.220 in 2007 to 0.337 in 2019, and even decreased in the 2010–2012 and the 2014–2015 periods. But it is the only efficiency with a rising trend in our research. Lastly, the lowest average efficiency through the time is the efficiency of commodity sales. It presents a gentle increment in 2007–2010, a fast decrease in 2010–2012, and a flat trend after 2012. The efficiency value was 0.193 in 2007, then increased to 0.224 in 2009, and kept around 0.140 from 2012 to 2019.

Based on the results of the above tables and figures, we have some further findings. First, nearly no HIDD goes beyond the others in the separate evaluation but the sequences of the HIDDs in the rankings are very different. Second, Nantong national HIDD ranks first in the all-in-total sales efficiency evaluation. This HIDD is also the top in the commodity sales efficiency evaluation. Hangzhou national HIDD is the champion of the technology sales efficiency and Ningbo national HIDD surpasses the others in the ranking of product sales efficiency. Third, only efficiencies of technology sales turnover change with time while the product, commodity and all-in-total sales turnover do not. These findings are important to support the view that the HIDDs improve their efficiency of technology sales when they are upgraded to the national list under the Torch initiative [52]. It supplements the current literature by finding that the HIDDs could promote innovation but their growth in total sales efficiency is not satisfying [16,22].

## 4 Conclusion

The evaluation of the efficiency of innovation clusters and zones has long been a hot topic in economic and societal growth. This article chose the Yangtze River Delta, the most technologically advanced region in China, as the study area to explain the economic efficiency of the national HIDDs. In this region, the number of national HIDDs increases from 9 in 2007 to 34 in 2019. Based on the stochastic frontier analysis, the study examines the impact of S&T spending and employment input on national HIDDs in the YRD's technology, product, commodity, and all-in-total sales turnover as the economic efficiency evaluation on the national HIDDs in the region.

The main findings are summarized as follows:

(1) The average efficiency trends of the all-in-total sales, product sales and commodity sales are decreasing while that of technology sales is increasing from 2007 to 2019. The average efficiency of all-in-total sales has been steadily declining for 13 years with only a slight increase in the 2007–2011 period. The average efficiency values of product sales, technology sales and commodity sales in HIDDs are in descending order. The average efficiency of product sales is almost stable with a gentle drop at the end, from 0.513 in 2007 to 0.500 in 2019. The average efficiency of technology sales turnover did not increase very fast from 0.220 in 2007 to 0.337 in 2019, and even decreased in the 2010–2012 and the 2014–2015 periods. The average efficiency of commodity sales presents a gentle increment in 2007–2010, a fast decrease in 2010–2012, and a flat trend after 2012.
(2) In the total sales efficiency evaluation, most early-enrolled HIDDs are in the high-efficiency group whereas the newcomers are in the low-efficiency group. The Nantong HIDD has the highest efficiency, followed by Wuxi, Taizhou, Suzhou, Nanjing, Shanghai ZJ, Xiaoshan, Ningbo, Suzhou IP and Jiangyin HIDDs. Huainan, Huaian, Tongling, Shanghai ZZ, Lianyungang and Suqian HIDDs rank very low in the total sales evaluation. An interesting finding is that two Shanghai HIDDs perform very different from each other. Besides, Hangzhou, Nantong and Ningbo HIDDs rank first in the technology, product and commodity sales efficiency evaluation correspondingly. In addition, the S&T labor and capital inputs have positive impacts on technology sales. An important finding is that the increase of S&T labor inputs would decrease the efficiency of the product, commodity and total sales while the increase of the S&T capital inputs is as expected a facilitator to the efficiency of product, commodity and total sales.

The findings have several policy implications. First, governments should formulate more policies to stimulate the increase of the overall sales efficiency. One important solution is to improve the structure of three types of efficiency because it is not relevant with the policy aim of the Torch initiative. Governments in the YRD region should continue to improve the efficiency of technology sales by designing attractive high-tech talent programs. Although this type of efficiency increases through the years, it is less than 0.4 on average. Governments could utilize our findings to see if a HIDD's original upgrading purpose has changed since it was upgraded to a national innovation cluster. Second, the newly enrolled HIDDs are easily rated low in the evaluation. Policymakers in these HIDDs should consider the degree of maturity of the innovation system by improving the investment environment. These HIDDs are often on the fringe of the YRD region, including HIDDs in north Jiangsu and Anhui. Nantong is a high-efficiency case of the newly-enlisted HIDDs. It reveals that the newly enlisted HIDDs have an opportunity of displacing the long-serving ones. Third, the S&T labor and capital inputs are not always effective in improving overall efficiency. The governments should be cautious about the S&T employment expansion. The S&T employment should focus on the high-quality talents rather than the quantity of the talents. The contribution of S&T labor to an innovation cluster is crucial, but the scale should also be considered. The total sales efficiency could be hampered by an extremely large amount of S&T labor input.

The study contributes to the literature in two aspects. First, this study contributes to the discussion on the role of political intervention in technological innovation by breaking down the economic efficiency into three major parts, the efficiency of technology, product and commodity sales. Although some research argues that political intervention leads to a lack of innovation [21], the findings could prove that the Chinese government's S&T initiative is beneficial to the increase of technology sales efficiency. Our findings support the view held by many other researchers [17,19,25]. Second, this study extends the economic efficiency evaluation on the innovation clusters by considering the S&T policy initiatives. This innovative design could help the policymakers to understand how the policy inputs are relevant with the different types of outputs. This innovative perspective also expands the previous efficiency evaluation in the high-tech zones in China [12,43,44,49].

This study still has limitations for future research. First, the limitation could be from the selection of the indicator. This research explains the economic efficiency from the perspective of S&T inputs and outputs, rather than the comprehensive efficiency. Future research could incorporate more variables to fix this issue. Second, the findings are limited to the HIDDs in the Yangtze River Delta. Future research could compare the findings in the YRD with those in the other regions.

## Data availability

The data in this study are available from the corresponding author upon request.

## CRediT authorship contribution statement

**Chao Yang:** Writing – review & editing, Writing – original draft, Software, Methodology, Funding acquisition, Formal analysis, Conceptualization.

## Declaration of competing interest

The authors declare the following financial interests/personal relationships which may be considered as potential competing interests:Chao Yang reports financial support was provided by 10.13039/501100010011Jiangsu Postdoctoral Research Foundation. Chao Yang reports financial support was provided by Jiangsu Social Science Foundation for Young Scholars. Chao Yang reports financial support was provided by National Social Science Fund of China. If there are other authors, they declare that they have no known competing financial interests or personal relationships that could have appeared to influence the work reported in this paper.
