# Supplementary material for: Evaluating economic efficiency of the national high-tech industrial development districts in the Yangtze River Delta by stochastic frontier analysis
Source: Heliyon. 2024 Apr 20;10(9):e30128. doi: 10.1016/j.heliyon.2024.e30128 (PMC11061737; doi:10.1016/j.heliyon.2024.e30128)
Supplement: Multimedia component 1 [file mmc1.docx]

Appendix A1: Table A1

Descriptive Statistics of the Data

| HIDD name | OutputT | | OutputP | | OutputC | | OutputA | | Labour | | Capital | |
| --- | --- | --- | --- | --- | --- | --- | --- | --- | --- | --- | --- | --- |
|  | Mean | St.dev | Mean | St.dev | Mean | St.dev | Mean | St.dev | Mean | St.dev | Mean | St.dev |
| Shanghai ZJ | 144,797,512 | 148,054,886 | 889,335,300 | 503,116,801 | 58,457,796 | 49,945,540 | 1,092,590,607 | 680,917,958 | 197,382 | 121,091 | 60,785,111 | 43,117,372 |
| Shanghai ZZ | 8,608,797 | 6,184,025 | 27,535,166 | 9,411,045 | 2,562,196 | 2,342,855 | 38,706,159 | 16,910,919 | 8,190 | 2,624 | 3,526,670 | 1,791,984 |
| Nanjing | 16,159,739 | 16,133,852 | 364,516,756 | 122,926,582 | 15,965,041 | 6,181,678 | 396,641,537 | 141,883,732 | 57,448 | 24,901 | 12,914,227 | 8,069,376 |
| Wuxi | 6,712,141 | 6,735,843 | 303,548,159 | 55,191,810 | 2,040,557 | 1,531,907 | 312,300,857 | 61,075,873 | 33,001 | 7,827 | 7,510,022 | 2,411,580 |
| Jiangyin | 2,890,894 | 6,265,953 | 129,542,723 | 23,715,498 | 2,161,377 | 2,763,626 | 134,594,995 | 24,782,339 | 9,702 | 2,213 | 3,544,554 | 1,383,657 |
| Xuzhou | 1,238,128 | 2,046,472 | 74,442,965 | 28,089,186 | 796,241 | 1,897,330 | 76,477,335 | 26,441,912 | 6,340 | 1,411 | 1,594,329 | 743,676 |
| Changzhou | 18,561,387 | 22,739,184 | 170,256,641 | 49,613,432 | 1,957,865 | 1,836,167 | 190,775,894 | 64,021,220 | 22,007 | 4,256 | 4,968,931 | 1,602,274 |
| Wujin | 1,771,855 | 1,437,721 | 86,473,240 | 21,191,366 | 20,152,030 | 20,981,355 | 108,397,125 | 43,080,533 | 13,516 | 3,096 | 2,518,213 | 1,066,092 |
| Suzhou | 10,676,565 | 4,300,003 | 247,138,567 | 50,002,346 | 4,984,847 | 5,248,152 | 262,799,979 | 56,646,059 | 31,797 | 7,230 | 6,413,106 | 2,327,828 |
| Kunshan | 1,016,160 | 1,069,644 | 155,438,921 | 35,379,668 | 2,882,659 | 2,890,208 | 159,337,741 | 36,140,943 | 19,935 | 5,705 | 3,819,016 | 1,731,257 |
| Suzhou IP | 15,042,895 | 13,095,212 | 335,021,359 | 59,907,536 | 14,216,614 | 12,393,125 | 364,280,868 | 83,192,624 | 59,034 | 18,654 | 13,470,849 | 7,098,550 |
| Changshu | 1,394,891 | 305,069 | 95,166,924 | 13,339,070 | 792,062 | 214,743 | 97,353,877 | 13,790,670 | 6,353 | 1,478 | 2,362,002 | 927,974 |
| Nantong | 4,845,477 | 1,586,473 | 181,729,100 | 70,229,106 | 3,904,679 | 2,192,370 | 190,479,255 | 73,572,353 | 8,089 | 1,078 | 4,599,348 | 990,255 |
| Lianyungang | 214,914 | 148,367 | 49,837,775 | 11,738,937 | 409,681 | 227,806 | 50,462,371 | 11,911,928 | 7,667 | 1,040 | 4,136,716 | 1,962,341 |
| Huaian | 73,073 | 97,497 | 18,767,257 | 4,039,384 | 246,975 | 201,090 | 19,087,305 | 3,760,101 | 2,525 | 245 | 687,895 | 113,193 |
| Yancheng | 519,296 | 521,055 | 49,966,040 | 11,386,001 | 1,823,489 | 1,049,382 | 52,308,825 | 10,982,374 | 4,157 | 673 | 942,574 | 205,989 |
| Yangzhou | 634,421 | 455,169 | 41,388,779 | 5,748,688 | 79,667 | 91,738 | 42,102,867 | 5,567,142 | 5,776 | 1,233 | 1,188,747 | 498,966 |
| Zhenjiang | 960,751 | 594,059 | 44,001,205 | 21,002,197 | 2,194,381 | 2,076,229 | 47,156,338 | 22,728,705 | 6,541 | 3,466 | 1,403,833 | 892,409 |
| Taizhou | 901,990 | 865,422 | 77,208,439 | 24,983,769 | 3,148,480 | 1,101,759 | 81,258,908 | 26,566,974 | 3,549 | 917 | 1,166,657 | 589,137 |
| Suqian | 165,885 | 80,546 | 27,994,998 | 4,771,618 | 2,143,290 | 1,589,721 | 30,304,173 | 3,457,823 | 3,013 | 753 | 774,924 | 235,644 |
| Hangzhou | 92,052,830 | 58,187,716 | 209,123,754 | 116,087,038 | 23,002,106 | 14,935,891 | 324,178,690 | 188,689,349 | 73,988 | 32,459 | 20,149,237 | 14,502,373 |
| Xiaoshan | 1,248,027 | 1,520,904 | 147,797,049 | 90,281,397 | 3,634,426 | 5,962,664 | 152,679,502 | 97,637,654 | 9,549 | 8,264 | 2,794,337 | 2,768,003 |
| Ningbo | 13,941,105 | 6,921,547 | 134,126,379 | 92,926,789 | 60,439,474 | 37,497,598 | 208,506,958 | 126,588,222 | 19,643 | 14,052 | 4,975,956 | 3,955,959 |
| Wenzhou | 1,086,284 | 1,363,228 | 46,844,945 | 14,357,987 | 421,491 | 445,335 | 48,352,719 | 15,610,003 | 8,346 | 4,524 | 1,366,230 | 954,920 |
| Jiaxing | 410,438 | 45,470 | 56,319,566 | 11,355,451 | 2,819,981 | 2,367,915 | 59,549,985 | 13,534,486 | 6,742 | 794 | 1,674,589 | 463,795 |
| Moganshan | 741,372 | 528,314 | 43,338,888 | 12,301,079 | 1,709,179 | 1,072,359 | 45,789,439 | 13,645,922 | 5,134 | 1,752 | 1,059,334 | 492,556 |
| Shaoxing | 814,641 | 1,847,906 | 37,047,350 | 24,873,523 | 676,299 | 1,010,331 | 38,538,289 | 26,398,724 | 4,237 | 3,586 | 927,026 | 1,003,677 |
| Quzhou | 119,093 | 62,266 | 72,517,188 | 18,827,818 | 628,558 | 335,914 | 73,264,839 | 19,093,546 | 7,792 | 1,960 | 1,607,769 | 775,106 |
| Hefei | 48,787,577 | 46,425,708 | 230,414,722 | 124,990,818 | 4,049,223 | 3,546,573 | 283,251,522 | 171,145,266 | 51,908 | 28,025 | 13,130,946 | 9,786,977 |
| Wuhu | 1,160,062 | 802,370 | 92,424,217 | 35,302,750 | 3,162,111 | 3,696,021 | 96,746,390 | 35,279,986 | 13,622 | 6,478 | 3,553,455 | 2,093,022 |
| Bengbu | 1,415,569 | 1,111,943 | 79,703,358 | 31,557,523 | 1,373,731 | 2,627,310 | 82,492,658 | 33,850,872 | 10,846 | 1,949 | 2,159,536 | 580,963 |
| Huainan | 152,381 | 51,921 | 10,356,832 | 9,588,994 | 204,948 | 118,073 | 10,714,160 | 9,758,988 | 1,393 | 545 | 181,769 | 83,960 |
| Maanshan | 5,937,073 | 6,738,155 | 72,512,288 | 18,089,810 | 699,503 | 502,877 | 79,148,864 | 24,218,331 | 5,995 | 761 | 2,145,351 | 663,888 |
| Tongling | 171,019 | 87,393 | 16,397,973 | 13,727,009 | 786,459 | 886,746 | 17,355,451 | 13,348,079 | 1,678 | 99 | 385,471 | 71,807 |

Source: China Torch Statistical Yearbooks.
